# Update on the Management of Non-Obstructive Azoospermia: Current Evidence and Unmet Needs

**DOI:** 10.3390/jcm11010062

**Published:** 2021-12-23

**Authors:** Ettore Caroppo, Giovanni M. Colpi

**Affiliations:** 1 Andrology Outpatients Clinic, Asl Bari, PTA “F Jaia”, Conversano, 70014 Bari, Italy; 2 Andrology Unit, Procrea Institute, 6900 Lugano, Switzerland; gmcolpi@yahoo.com

Azoospermia, defined as the absence of sperm in the ejaculate after examination of the centrifuged specimens, affects about 1% of the male population and 10–15% of infertile men. In about two-thirds of cases, this is caused by severe spermatogenic dysfunction [1], and it is commonly termed “nonobstructive azoospermia” (NOA) to differentiate it from the less severe form of azoospermia caused by the obstruction of the seminal tract (obstructive azoospermia—OA); the latter affects the remaining one-third of cases. Managing patients with NOA is challenging due to the severity of spermatogenic dysfunction and the lack of medical treatments, with surgical retrieval of testicular sperm being the only way of enabling some of these patients to father their own biological children. In-depth clinical knowledge is key for supporting clinical reasoning and decision making when counselling patients with NOA, and surgical skill is required to maximize the outcome of surgical procedures that aim to retrieve testicular sperm. Therefore, the present Special Issue was designed to provide young reproductive urologists and endocrinologists with an update of the scientific evidence in the field, together with surgical tips.

The differential diagnosis between OA and NOA is mandatory for the correct management of patients; men with OA have intact spermatogenesis, so that sperm may be surgically retrieved in the vast majority of cases by means of minimally invasive techniques [2]. Sperm retrieval is successful in no more than 58% of men with NOA, provided that the most effective surgical technique, namely, microdissection testicular sperm extraction (mTESE), is used [3]. In the first article of the present Special Issue, Danilo L. Andrade, Marina C. Viana and Sandro C. Esteves showed that the differential diagnosis between OA and NOA may be effectively accomplished in most patients by means of a standardized male infertility workup, which should include a detailed medical history, a careful physical examination with a focus on secondary sexual characteristics, a semen analysis obtained on at least two occasions and assessed according to the World Health Organization, hormonal evaluation (serum FSH, LH, prolactin and testosterone levels), genetic tests (karyotype and Y chromosome microdeletion analysis, screening for cystic fibrosis transmembrane conductance regulator gene mutations), and a scrotal and transrectal ultrasound, with testis biopsy being reserved only for the cases of doubt [4].

Genetic tests are useful for diagnostic and prognostic purposes in men with NOA. Csilla Krausz and Francesca Cioppi reviewed the most common genetic abnormalities found in men with NOA and illustrated their possible consequences on their general and reproductive health, as well as on their children’s health. They also dedicated a chapter to the conflicting evidence regarding health issues in offspring conceived by ICSI with testicular sperm retrieved in patients with NOA, and highlighted the potential diagnostic utility of performing whole-exome sequencing in men with NOA due to meiotic arrest [5].

Management of men with NOA would undoubtedly benefit from the identification of clinical and laboratory markers of spermatogenesis able to individuate those patients really suited for mTESE. The evidence from the literature in this field, reviewed by our group for this Special Issue [6], clearly shows that, although few factors, including complete AZFc deletion or history of cryptorchidism, were associated with better chances of successful sperm retrieval (SSR), no clinical or laboratory marker is able to predict the outcome of mTESE, due to the anatomic singularity of the testes of men with NOA, which may hide few loci of spermatogenesis. Moreover, the great impact of the surgeon’s skill and experience, together with the time and efforts dedicated to the search for sperm in the testicular specimens, may have an impact on mTESE outcome. Promising results arising from studies investigating the predictive ability of molecular markers expressed in the seminal plasma should be confirmed by further studies.

Azoospermia due to spermatogenic dysfunction is an untreatable condition, apart from the rare cases of patients with hypogonadotropic hypogonadism. Nonetheless, a role for the hormonal stimulation of spermatogenesis to improve sperm retrieval rates in these patients was proposed by some authors. As summarized in our review paper, while the optimization of serum testosterone level seems to be justifiable in men with hypogonadism, the available evidence is insufficient for recommending hormonal treatment before surgery in men with NOA [7].

The introduction of mTESE in 1999 greatly improved the chance of retrieving testicular sperm in patients with NOA, by enabling the identification of foci of spermatogenesis at high magnification even in patients with nearly atrophic testes. In the two review papers coauthored with Nahid Punjani and Caroline Kang, the pioneer of this surgical technique, Prof. Peter N. Schlegel, illustrates how to manage patients with NOA and optimize the success of mTESE [8], and sheds light on the reproductive chances of men with NOA according to the underlying etiologies (Klinefelter syndrome, Y chromosome microdeletions, chemotherapy-associated NOA, cryptorchidism) [9]. Both papers are a must read for reproductive urologists.

A learning curve is required to improve the outcome of mTESE. A detailed description of mTESE surgical procedures, accompanied by an extensive iconography, is provided by a review authored by our group, in view of the vast surgical experience in this field of our leading urologist [10].

The outcome of mTESE is greatly affected by the accuracy in testicular sperm processing techniques. Kaan Aydos and Oya Sena Aydos reviewed the available sperm selection procedures, as well as the different approaches to testicular sperm cryopreservation, providing valuable suggestions for embryologists and clinicians about how to effectively handle testicular specimens and testicular sperm to maximize the outcome of mTESE [11].

The laboratory techniques used for testicular sperm processing are highly labor-intensive and subject to inter-laboratory and intra-laboratory variability. In view of his pioneering studies in the field of microfluidic technology applied to gamete and embryo isolation and culture, Gary D. Smith, joined by Clementina Cantatore and Dana A. Ohl, analyzed the potential utility, benefits, and shortcomings of such a technology to the isolation of non-motile sperm from retrieved NOA testicular samples [12].

Testicular surgery is not devoid of complications. Testicular damage is often a complication of conventional testicular sperm extraction (cTESE), as well as of testicular aspiration, while mTESE may be a more conservative surgical strategy, since it enables the identification of subalbuginal vessels and possibly avoids residual bleeding inside the tunica albuginea, which often results in testicular tissue damage. Still, both cTESE and mTESE may result in transient or, less frequent, permanent hypogonadism due to Leydig cells dysfunction. Evangelia Billa, George A Kanakis and Dimitrios G Goulis reviewed this interesting topic, explaining how hypogonadism may depend upon the underlying histology, the number of previous testicular surgeries, the etiology of NOA, and the size of the testes; in some patients, e.g., those with Klinefelter syndrome, the decrease in testosterone levels may be more profound and of longer duration [13].
